# Combined thermal manikin and thermal model predictions of working times in fully encapsulated impermeable suits

**DOI:** 10.1186/2046-7648-4-S1-A142

**Published:** 2015-09-14

**Authors:** Emiel A DenHartog, A Shawn Deaton

**Affiliations:** 1 Thermal Protection and Comfort Center (TPACC), College of Textiles, North Carolina State University, Raleigh, NC, USA

## Introduction

This project aimed to develop guidelines for safe working times in fully encapsulated impermeable suits and incorporate these data into an existing Chemical Companion Decision Support System (CCDSS) that is used by First responders in the US and abroad. The CCDSS provides guidelines on many operational aspects of response to a hazardous materials (HazMat). This study addresses the use of a thermos-physiological model, combined with a sweating thermal manikin, to simulate the data and compared that to the experimental data.

## Methods

Using 17 local male firefighters in an age range of 25 to 50, six commercially available suits were used, in combination with the self-contained breathing apparatus (SCBA). Three climates were evaluated in experiments lasting maximally 60 minutes: moderate (20°C WBGT), warm (30°C WBGT) and hot (37°C WBGT) at a moderate work load (229 W.m^-2^). Additionally, 2 workloads (low, 164 W.m^-2^and high, 283 W.m^-2^) were evaluated in the moderate climate. Measurements included gastro-intestinal (GI) temperature 4 local skin temperatures (ISO9886), body mass loss, heart rate and comfort scores. In total 163 tests were performed with 45 different subjects. In addition the suits were evaluated on a standard Sweating Thermal Manikin to which Thermoanalytics' RadTherm model was linked to simulate the thermo-physiological response to the manikin's heat loss. The model outputs are the predicted core temperature, skin temperature and sweat rate over time; and the model also has the possibility to show differences between simulated hypothalamus and rectal temperatures.

## Results

The results show a wide range of working times, decreasing with temperature from a median of 50 minutes (90% range 30 - 60 minutes) in the moderate climate to a median of 28 minutes (90% range 18 - 34 minutes) in the hot climate. Similarly the higher work load reduced working time to a median of 32 minutes (90% range 18 - 46 minutes), in the moderate climate whereas the median work time was the full 60 minutes (13 out of 16 completed 60 minutes) in the low work load. The Manikin, combined with the Thermoanalytics physiological model showed very similar prediction curves for the core temperature, but the hypothalamus predicted a more rapid increase of the body temperature, mostly below 10% of the range of the human subject responses, whereas the simulated rectal temperature lagged the measured GI pill temperatures, mostly above 90% of the range of the human subject responses.

## Discussion and conclusion

The temperature measured by the pill most closely reflects a rectal temperature which has shown to be lagging compared to central temperature. In the latest version the Thermoanalytics model intends to predict both Hypothalamus, reflecting central temperature and rectal temperature. The data presented here suggested that the actual measured data had a faster response than the rectal temperature simulation. As insufficient data was available on the exact calculation of the thermal inertia and the local heat and mass transfer through the blood flow, it was inconclusive if the model was under-predicting the thermal response or the actual pill temperature exhibited a true middle value between rectal and hypothalamus temperature. For prediction of safe working times, based on estimated climate and work loads, the Thermophysiological model linked to the thermal manikin may be very helpful, but further improvements to the time lags as measured rectal and by the temperature pill may be needed.

